# A Density Clustering RAPID Based on an Array-Compensated Damage Index for Quantitative Damage Diagnosis

**DOI:** 10.3390/s24154904

**Published:** 2024-07-29

**Authors:** Qiao Bao, Tian Xie, Yan Zhuang, Qiang Wang

**Affiliations:** College of Automation and College of Artificial Intelligence, Nanjing University of Posts and Telecommunications, Nanjing 210023, China; 1022051314@njupt.edu.cn (T.X.); 1222056525@njupt.edu.cn (Y.Z.); wangqiang@njupt.edu.cn (Q.W.)

**Keywords:** guided waves, RAPID, density clustering, structural health monitoring

## Abstract

Guided wave array-based structural health monitoring (SHM) is a promising solution for diagnosing damage in metal-connected structures. In this field, the reconstruction algorithm for probabilistic inspection (RAPID) is one of the most widely used algorithms for performing damage localization. In this paper, a density clustering RAPID based on an array-compensated damage index is proposed. A new probability distribution function was constructed based on a new damage index, which is adaptive to different elements in the sensor array to compensate for performance variation. Then, the imaging matrix of the RAPID algorithm was density-clustered to obtain the location and degree of damage. Finally, the method was verified by experiments on a stiffened aluminum plate. The experimental results demonstrate that the method achieves damage localization and enables quantitative damage diagnosis.

## 1. Introduction

During long-term service, metal-connected structures may fail due to loose bolts, crack expansion, etc. Thus, monitoring the location and degree of damage in metal-connected structures is important. Structural health monitoring (SHM) based on a guided wave array is one of the most widely used structural damage detection technologies [1]. Guided waves are characterized by their slow energy attenuation, extensive propagation distances, high sensitivity to structural changes, and sufficient detection range [2]. When guided waves encounter damaged areas within a structure, they undergo reflection and scattering, which causes the waves to carry information about the damage. By analyzing a received guided wave, damage within a structure can be identified and evaluated [3].

Since array-based damage imaging is visualized and able to enhance the signal–noise ratio, researchers have performed a large amount of research on damage imaging technology based on guided waves. At present, several mature imaging techniques include the delay–sum algorithm [4], the phased array imaging algorithm [5], the time-reversal algorithm [6], the synthetic aperture focusing technique [7], the computed tomography algorithm [8], Lamb wave minimum variance imaging [9], the MUSIC technique [10], the spatial-wavenumber filter technique [11], and the reconstruction algorithm for probabilistic inspection (RAPID) [12].

Among these methods, the RAPID algorithm has been the focus of many researchers because of its simple principles, fast operation, and accurate damage localization. The RAPID algorithm compares signals obtained under healthy conditions with current monitoring signals, and the difference between the signals can be represented by a damage index (DI). Then, damage imaging of the structure can be properly constructed through the combined effects of damage indexes and damage probabilities for each sensing path.

Extracting accurate differences between signals is crucial for the RAPID algorithm. Wang et al. [13] estimated the probability of the existence of damage on an aluminum plate by using the correlation coefficient between a damaged signal and an undamaged signal. Zhao et al. [14] discussed the effectiveness of the signal correlation function and proved that the correlation function can detect damage even in the presence of noise. Hettler et al. [15] used the baseline-dependent and the baseline-independent methods to optimize RAPID and monitor the impact of damage on composite structures. Jin et al. [16] combined a variety of signal correlation calculation functions in the time domain and frequency domain into a damage index, which effectively improved the accuracy of the RAPID algorithm. Wang et al. [17,18] introduced Shannon entropy and digital damage fingerprint to reduce errors in the damage index obtained from signal correlation comparison, and accurately identified damage to stiffeners under an environment of variable temperature. Huo et al. [19] proposed an integrated method of the elliptic trajectory method and the RAPID algorithm based on the Bayes framework, which successfully integrated multiple damage indexes and verified the reliability of the algorithm on composite plates. Therefore, most subsequent studies calculate the correlation between undamaged signals and damaged signals to obtain a damage index, and Li et al. [20] studied the robustness of the RAPID algorithm with a signal correlation function to monitor a board-like structure.

The probability distribution function of the RAPID algorithm varies linearly according to the damage index and the distance between the detected point and the sensing path. In other words, regardless of whether the actual damage is located on the sensing path, the value of probability on the sensing path is always the greatest. To minimize the impact of this issue, Zhou et al. [21] used elliptical probability distribution to optimize the distribution function and proved that the RAPID method can quickly locate damage in the structure under examination. The effectiveness of the algorithm has been verified by successfully reconstructing the probability imaging of damage on an aircraft wing through experiments. Moreover, Zhao et al. [22] compared several tomography technologies, such as the filter back projection algorithm, algebraic reconstruction technology and the RAPID algorithm, and concluded that the RAPID algorithm has flexibility in array geometry selection and can completely automatically reconstruct imaging in good quality. Lee et al. [23] evaluated the effect of the effective area of elliptical probability distribution, which depends on the selection of the scaling parameter [24]. However, fixing the scaling parameter can lead to distortion in damage imaging. To address this issue, an adaptive parameter algorithm based on a variable form factor by considering the localization of the damage is proposed [25,26,27]. The use of the variable shape factor method can significantly enhance the reconstruction of damage imaging. In addition to the scaling parameter, the frequency of excitation waves [28], the effective area of the sensor array [29,30,31], the length of signal sampling [32], and signal processing algorithms [33] also have an impact on the RAPID algorithm.

The above scholars have achieved accurate damage localization by enhancing damage indexed through signal processing techniques and optimizing the probability distribution function in conjunction with other methods. However, firstly, given the escalating demand for damage detection in engineering applications, relying on a single damage localization method is insufficient to meet actual needs. Secondly, because of the varied performance of different elements in the sensor array, the same damage can cause varied signal amplitudes and phase changes for every sensing path, even when the propagation distance and direction are exactly the same. In addition, the RAPID algorithm’s construction of identical probability ellipses for each sensing path will cause excessively large damage probability values along the direct sensing paths and at the overlapping regions of the ellipses, which can result in artifacts and distorted damage shapes. This makes it challenging to accurately extract damage information from the image.

To address these issues, we propose a density clustering RAPID based on an array-compensated damage index. Firstly, to overcome the limitations of relying on a single signal feature, the proposed method extracts and integrates multiple features to derive a comprehensive damage index. Then, an array-compensated damage index is calculated using SWT (Shannon wavelet transform). Subsequently, the algorithm is enhanced by incorporating the distance between the damage localization and the sensing path, based on the magnitude of the ADI. This enhancement, when integrated with the density clustering algorithm, effectively eliminates false damage probability values. Finally, the experimental results on an aluminum plate show that the method proposed in this paper can accurately determine damage localization and enable quantitative damage diagnosis.

## 2. The Density Clustering RAPID Algorithm with an Array-Compensated Damage Index

This paper proposes a density clustering RAPID with an array-compensated damage index. In this section, we propose an array-compensated damage index (ADI) to account for signal variation due to the varied performance of different elements in the sensor array. Then, a new probability distribution function is obtained through the ADI (array-compensated damage index). Finally, the damage probability matrix is refined using the principle of density clustering.

### 2.1. The Principle of the Original RAPID Algorithm

**Figure 1:**
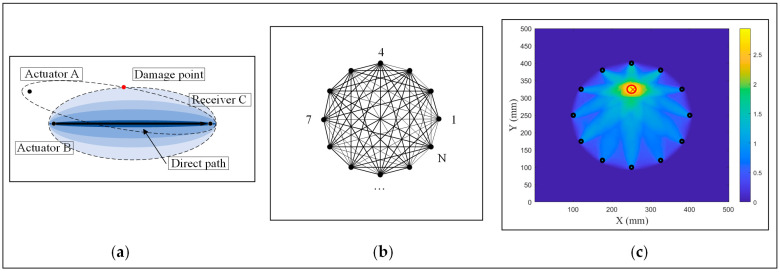
Models of the RAPID algorithm: (**a**) the elliptical weight of a single sensing path; (**b**) the sensing paths of circular sensor array; (**c**) the probabilistic inspection of damage.

To achieve damage imaging based on the RAPID algorithm, the signal features of each sensing path are first obtained by comparing actual measured signals with a reference signal, i.e., the DI. Then, the damage probability around this sensing path can be calculated based on a proper probability distribution function, as shown in Figure 1a. Usually, the probability distribution function adopts an elliptical weight function, expressed as Equations (1) and (2) [12,14]:

(1) $$W_{k}=\left\{\begin{array}{cc} \frac{1-R_{k}\left(x,y\right)}{\beta -1}, & R_{k}\left(x,y\right)<\beta \\ 0, & R_{k}\left(x,y\right)\geq \beta \end{array}\right.$$

(2) $$R_{k}\left(x,y\right)=\frac{\sqrt{{\left(x-x_{k}^{a}\right)}^{2}+{\left(y-y_{k}^{a}\right)}^{2}}+\sqrt{{\left(x-x_{k}^{r}\right)}^{2}+{\left(y-y_{k}^{r}\right)}^{2}}}{\sqrt{{\left(x_{k}^{a}-x_{k}^{r}\right)}^{2}+{\left(y_{k}^{a}-y_{k}^{r}\right)}^{2}}}\;$$

where $\left(x,y\right)$ is the coordinate of the detected point, $\left(x_{k}^{a},y_{k}^{a}\right)$ and $\left(x_{k}^{r},y_{k}^{r}\right)$ are the coordinates of the actuator and the receiver in the k sensing path, a in the superscript stands for actuator and r in the superscript stands for receiver, and β is the shape factor for controlling the range of the elliptical weight function [14]. If the measured point (x,y) is on the central axis of the ellipse, $R_{k}\left(x,y\right)=1$ and $W_{k}=1$. If the measured point (x,y) is on the boundary of the maximum effective ellipse or outside the ellipse, $R_{k}\left(x,y\right)=\beta$ and $W_{k}=0$.

The RAPID algorithm usually uses a sensor array to obtain the comprehensive signal features of the measured structure, such as a circular sensor array, known for its comprehensive detection capabilities and minimal error [31]. Therefore, a circular sensor array is used in this paper. A circular sensor array with N elements is able to form N·(N−1)/2 sensing paths, as shown in Figure 1b. Then, by exciting each sensing path in a cyclic manner, the damage imaging matrix from each path is combined and overlaid to identify the damage location [34,35]. The corresponding formula is expressed as Equation (3):

(3) $$P\left(x,y\right)=\sum_{k=1}^{N\cdot (N-1)/2}{DI}_{k}\cdot W_{k}$$

Finally, probabilistic inspection of the damage can be reconstructed, as shown in Figure 1c.

### 2.2. The Damage Index Compensated by a Sensor Array

In this paper, to extract the damage information more accurately, nine damage indexes are selected to be calculated based on comparative analysis from Moix-Bonet’s prior research, as detailed in Table 1.

The sensitivities of different damage indexes to damage are different. Thus, the superposition method is used to combine nine methods to calculate DIs to make up for their shortcomings [37], expressed as Equation (4):

(4) $$DI=\sum_{i=1}^{I}{DI}^{i}$$

However, in practical applications, the same damage can cause varying signal changes for every sensing path, caused by the different performance of each element in the sensor array, even when the propagation distance and direction are exactly the same. The following two reasons can mainly explain this deviation. First, there is a slight performance difference in the PZT itself. Second, it is challenging to maintain the same rubber layer thicknesses of all PZTs, which affects the electromechanical coefficient [38], resulting in an amplitude difference for each sensor signal.

**Figure 2:**
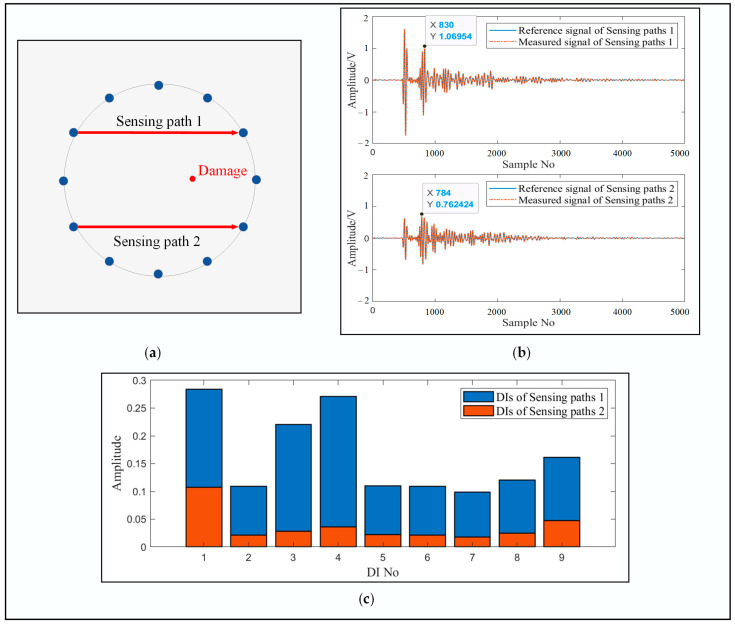
The signals obtained by the sensors under the same conditions: (**a**) the selected sensing paths in the circular sensor array; (**b**) the signals of the selected sensing paths; (**c**) the DIs of the selected sensing paths.

For example, the received guided waves from two sensing paths in a healthy state and a damaged state are plotted in Figure 2a. It can be seen that the propagation distances and directions of these two sensing paths are the same, as is the distance between the damage and these two sensing paths. Reference signals were collected before creating damage and measured signals obtained in the damaged state. But there exists an obvious difference between the amplitudes of the two groups of guided waves, i.e., 1 V and 0.7 V, respectively. The corresponding DIs are also plotted in Figure 2b. It can be surmised that the DI would be larger when the signal amplitude becomes higher. In this case, the damage imaging result would be shifted to the sensing path with a higher amplitude, thus leading to a localization error. To compensate for this kind of error, an array-compensated adaptive damage index (ADI) is proposed in this paper.

**Figure 3:**
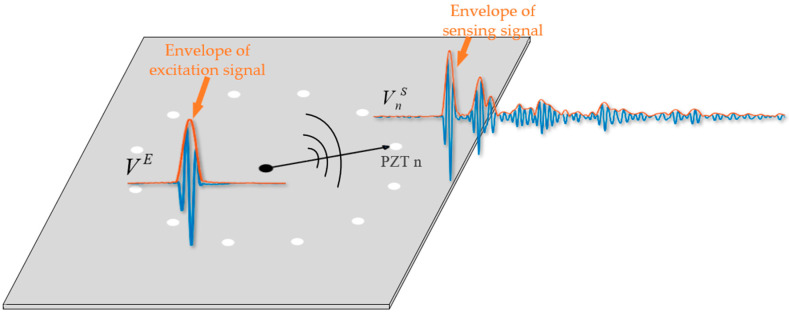
The schematic diagram of array compensation.

Firstly, a PZT is installed in the center of the circular sensor array, and the distance between this PZT and each element in the array are the same, as shown in Figure 3. The sensor in the center acts as the actuator and the sensor in the circular sensor array acts as the receiver to receive the sensing signal.

Considering that scattered waves will convey structure and damage information, the amplitude of a direct wave is mainly related to the electromechanical coefficient of the PZT in each sensing path. Therefore, the compensation coefficient of each PZT is derived by inverse calculation of direct wave amplitude. The envelopes of the excitation signal and sensing signal are extracted by Shannon wavelet transform [39]. Thus, the amplitude of the excitation signal can be obtained, as can the amplitude of the direct wave of the sensing signal received by PZT n, denoted as $V^{E}$ and $V_{n}^{S}$, respectively. The compensation coefficient corresponding to PZT n can be calculated as Equation (5):

(5) $$m_{n}=\frac{V_{n}^{S}}{V^{E}}$$

According to Equation (5), the larger the $V_{S}$ amplitude, the larger $m_{n}$ will be. Therefore, the result is normalized and reversed as the weight of the PZT, and the DI is compensated.

(6) $${ADI}_{k}=\frac{1}{m_{a}m_{r}}{DI}_{k}$$

where k denotes the $k^{th}$ sensing path. a in the superscript stands for the actuator number and r in the superscript stands for the receiver number corresponding to the $k^{th}$ sensing path, respectively.

### 2.3. Optimizing Probability Distribution Function Based on ADI

The original RAPID algorithm employs a fixed shape factor β, which leads to an overestimation of the probability value along the sensing path and within the overlapping regions of the probability ellipse. This overestimation can distort the detected damage and introduce artifacts into the imaging process. To address this issue, this paper constructs a new shape parameter $\beta^{*}$ for each path, which is related to the ADI of a corresponding sensing path, expressed as Equation (7):

(7) $$\beta_{k}^{*}=\beta -\alpha \cdot \frac{{ADI}_{k}-min(ADI)}{\max\left(ADI\right)-min(ADI)}$$

where k is the kth sensing path, $\beta_{k}^{*}$ controls the maximum area of the effective ellipse, β is the fixed shape factor obtained from previous experiments, and α is the constraint coefficient (the larger α is, the more sensitive $\beta_{k}^{*}$ is according to the change in the ADI).

Incorporating the new shape factor $\beta_{k}^{*}$ into Equation (1) yields a new probability distribution function, as shown in Equation (10):

(8) $$W_{k}\left(x,y\right)=\left\{\begin{array}{c} \frac{\beta_{k}^{*}-R_{k}\left(x,y\right)}{\beta_{k}-1};1<R_{k}\left(x,y\right)<\beta_{k}^{*}\\ \;0;\;R_{k}\left(x,y\right)\geq \beta_{k}^{*} \end{array}\right.$$

If the measured point (x,y) is on the central axis of the ellipse, then the value of $R_{k}\left(x,y\right)$ is equal to 1. If the measured point (x,y) is on or outside the boundary of the largest effective ellipse, then $R_{k}\left(x,y\right)=\beta_{k}^{*}$.

By combining Equations (6) and (8), the probability of damage can be obtained. The formula is as follows:

(9) $$P\left(x,y\right)=\left[A\begin{array}{ccc} DI(1) & ADI(2) & \begin{array}{cc} \cdots & ADI(N\cdot (N-1)/2) \end{array} \end{array}\right]\cdot \left[\begin{array}{c} W_{1}(x,y)\\ \begin{array}{c} W_{2}(x,y)\\ \vdots \end{array}\\ W_{N\cdot (N-1)/2}(x,y) \end{array}\right]$$

### 2.4. Density Clustering for Quantitative Damage Diagnosis

The RAPID algorithm’s propensity to overlap probability ellipses near the sensor can lead to points with false damage values. Directly screening out false damage points based on damage probability may ignore the real damage near the sensor. Therefore, the density clustering algorithm is employed to filter out these false damage points. This clustering algorithm identifies regions of high density within the damage probability matrix, which usually indicate actual damaged areas or structural anomalies. When the number of points within a neighborhood radius *R*, which exceeds the specified minimum threshold, is greater than a specified value (MinPts), the area is identified as a damaged region.

(10) $$Cond=\{\left(x,y\right)\in P|P\left(x_{i}^{R},y_{i}^{R}\right)\geq threshold\}$$

(11) $$TP\left(x,y\right)=\left\{\begin{array}{c} P(x,y);Cond\geq MinPts\\ 0\;;Cond<MinPts \end{array}\right.\;$$

where $\left(x,y\right)$ is the core point, $\left(x_{i}^{R},y_{i}^{R}\right)$ is the $i^{th}$ point in the R range of $\left(x,y\right)$, Cond counts the number of ${(x}^{R},y^{R})$ that satisfy the condition, and MinPts defines the number of points in the neighborhood required for a core point.

A damage image of the measured structure is obtained by the method proposed in this paper. The point with the largest probability of damage is taken as the damage location result of the algorithm, that is $\left(\hat{x},\hat{y}\right)$. The formula is as follows:

(12) $$\left(\hat{x},\hat{y})=argmax(TP\right)$$

**Figure 4:**
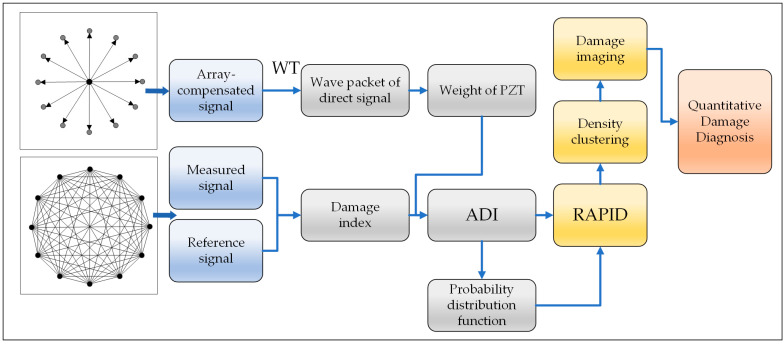
Diagram of the proposed method.

Based on the location of the damage, the number of pixels (NOP) in the damage image is used to quantify the degree of damage, which is the number of non-zero pixels in the matrix of the probability of damage. The diagram of the proposed method is shown in Figure 4.

## 3. Experimental Verification on Aluminum Plate

The density clustering RAPID based on an array-compensated damage index is verified through experimental verification. Experiments on the stiffened aluminum plate are conducted with varying degrees of damage to assess the method’s performance.

### 3.1. Experimental Setup

**Figure 5:**
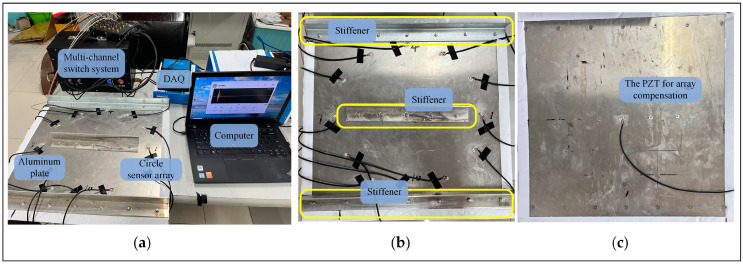
Instruments in the experiment: (**a**) the experimental instruments and aluminum plates; (**b**) the placement of sensors and aluminum stiffeners on the aluminum plates; (**c**) the arrangement of PZT13 used to compose the compensation array.

The experimental setup comprises an arbitrary waveform generator and data acquisition device, a multi-channel switching system, a computer, and a stiffened aluminum plate. The data acquisition device is utilized to generate an excitation signal and capture output signals from the sensor. The multi-channel switch system is used to switch different sensors on or off to achieve multi-channel scanning. A power amplifier and a charge amplifier are also integrated into the multi-channel switch system. The power amplifier is employed to boost the excitation signal, while the charge amplifier enhances the collected signals, as shown in Figure 5a. During the experiment, a three-period corrected sine wave with an amplitude of ±50 V is used as the excitation signal, and its center frequency was set to 50 kHz to generate guided waves dominated by the A0 mode. The sampling rate is set to 2 MSPS, and the sampling length is 5000.

A 500 mm × 500 mm × 2 mm aluminum plate, characterized by a density of 2700 kg/m^3^, Poisson’s ratio (v) of 0.33, and Young’s modulus (E) of 70 Gpa is used. The plate has three aluminum stiffeners secured by bolts, as shown in the yellow box in Figure 5b. The bolt holes are Ø6 mm. Twelve PZT-5A piezoelectric elements with a diameter of 8 mm and a thickness of 0.48 mm are arranged on a circular array with a diameter of 360 mm. The PZT located at (430 mm, 250 mm) is designated as sensor No. 1 (PZT1), and each subsequent sensor is named counterclockwise from PZT1 to PZT12. Since the stiffeners located at the center of the circular array, PZT13 is installed on the back of the aluminum plate to compensate for the sensor array at (250 mm, 250 mm), as shown in Figure 5c. For the panel structure, guided waves propagate between two parallel surfaces. Therefore, it is feasible to install PZTs on both the front and back surfaces of the structure for compensation.

**Figure 6:**
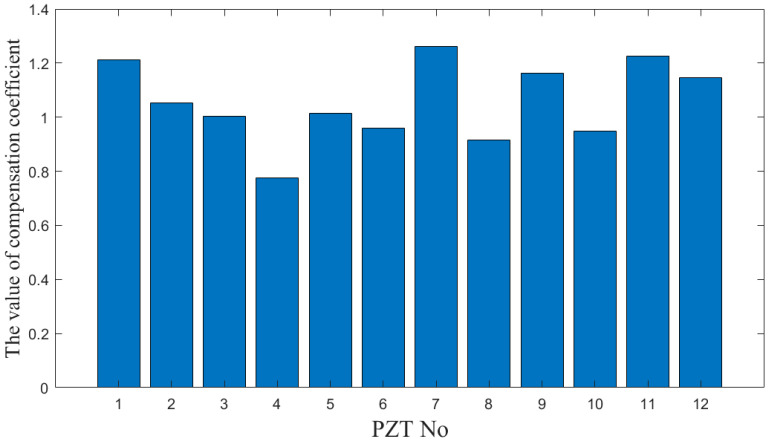
Compensation coefficient of each element in the sensor array.

The compensation coefficients of the 12 PZTs are obtained by processing the array-compensated signals, among which PZT7 exhibits the highest coefficient of 1.25. As shown in Figure 6, the compensation coefficients of PZT1, PZT7, and PZT11 are significantly higher than those of the other sensors, and all exceed 1.2; PZT4 demonstrates a notably lower compensation coefficient, below 0.8, while the compensation coefficients for the remaining sensors average around 1, signifying normal operation. Therefore, the ADI for the sensing paths, which include PZT1, PZT7, and PZT11, but exclude PZT4, will be significantly higher.

### 3.2. Typical Damage Scattered Signal

**Figure 7:**
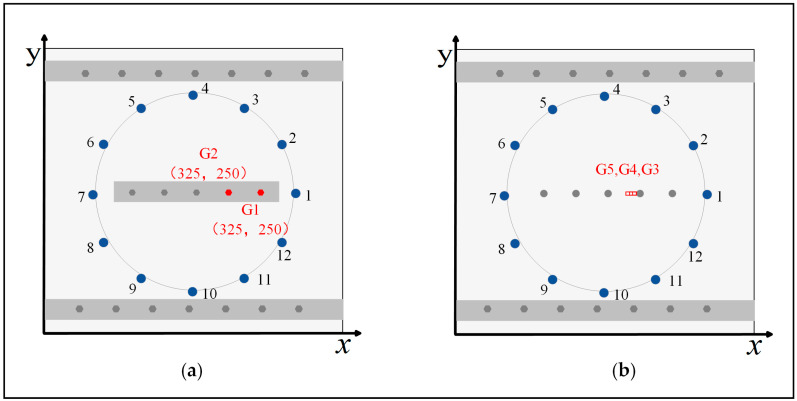
Actual positions of damage sites: (**a**) the loose bolts of the aluminum stiffener at G1 and G2; (**b**) the crack damage on the edge of the bolt holes at G3, G4, and G5.

In the experiment, five groups of damage are simulated in total. As shown in Figure 7a, the damage of G1 and G2 are simulated through the loose bolts of the aluminum stiffener, respectively. G3 is a crack on the edge of the bolt hole, and its length and width are 2 mm and 1.5 mm, respectively. During the experiment, G4 and G5 extended the crack of G3 to 4 mm and 6 mm, respectively, as shown in Figure 7b.

**Figure 8:**
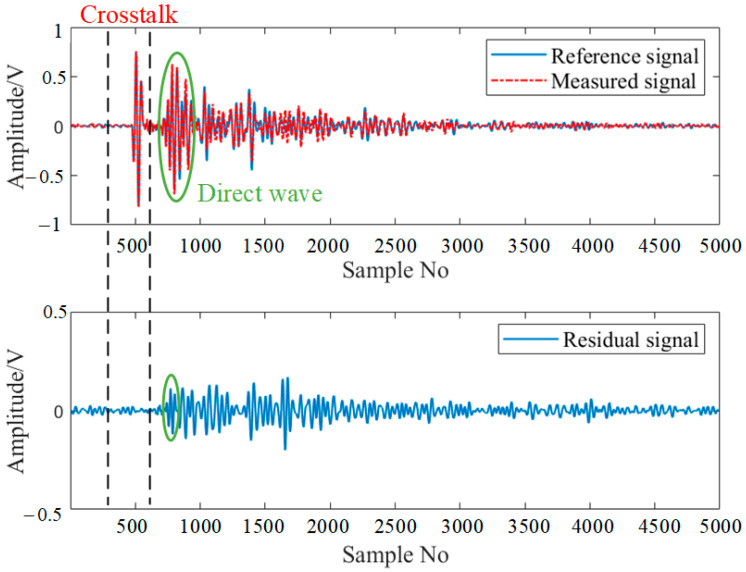
Typical damage-scattered signals of G1 corresponding to PZT3-PZT12.

Since 12 elements are actuated successively and other elements are used as the receiver, 66 sensing paths are scanned in total in the experiment. The signals captured from these sensing paths can be categorized into two distinct categories. The first category includes sensing paths that either directly pass through the damage or are in close proximity to it. For instance, the sensing path PZT3-PZT12 of G1 is depicted in Figure 8. The signal packet before the direct wave is the crosstalk [40]. A direct wave transformation is then observed, because the edge-reflected wave is interfered with by the measured structure; it passes through the actual signal on the damaged sensing path, resulting in significant amplitude reduction. The residual signal difference between the actual signal and the reference signal of PZT3-PZT12 is calculated, and the maximum amplitude of the amplified signal exceeds 0.2 V.

**Figure 9:**
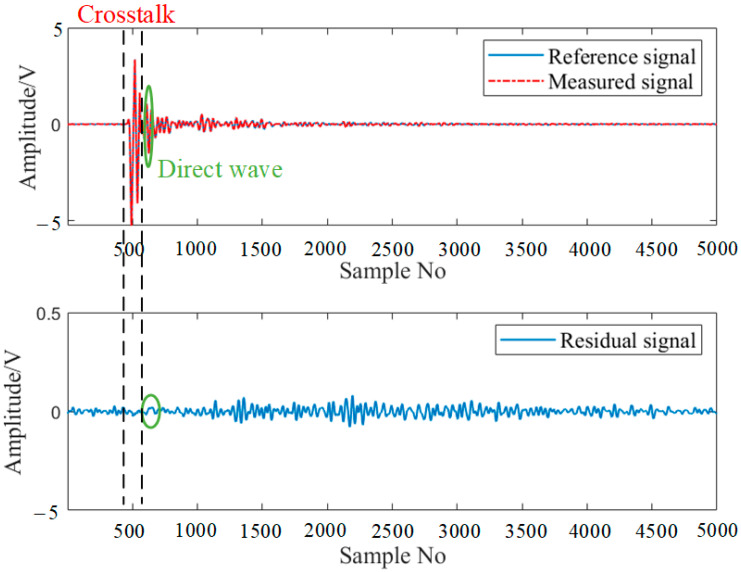
Typical damage-scattered signals of G1 corresponding to PZT6-PZT8.

The second category consists of sensing paths that are distant from the damage. For example, the guide wave corresponding to the sensing path of PZT6-PZT8 is shown in Figure 9. The amplitude of the residual signal of the selected signal segment is small. The maximum amplitude of the residual signal is less than 0.05 V. The small amplitude, when transformed into the ADI, results in a lower ADI value.

**Figure 10:**
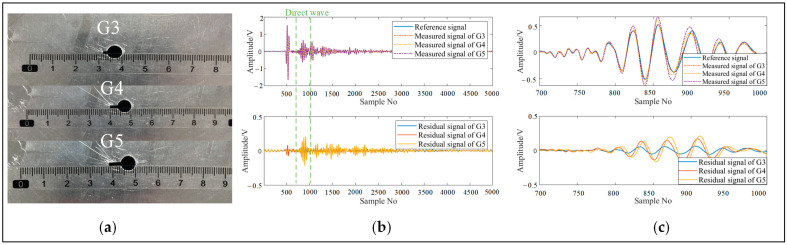
The expansion of cracks: (**a**) the real picture of cracks at G3, G4, and G5; (**b**) the reference signals and the measured signals of G3, G4, and G5; (**c**) the image of 700 to 1000 sampling points of the signals is enlarged.

Figure 10a shows a crack of 2 mm in G3, 4 mm in G4, and 6 mm in G5. Figure 10b shows the signal acquired by PZT3-PZT11 at a 2 mm crack, a 4 mm crack and a 6 mm crack. Figure 10c provides an enlarged image of the signal from 700 to 1000 sampling points, and it is clear that the residual signal gradually increases with the expansion of the crack, which will gradually increase the value the ADI.

**Figure 11:**
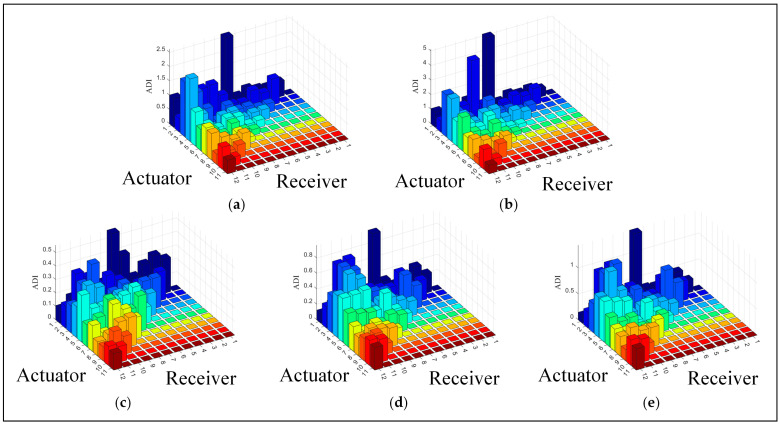
The damage index of five groups of damage compensated by sensor array: (**a**) G1; (**b**) G2; (**c**) G3; (**d**) G4; (**e**) G5.

The ADIs for two groups of loose bolts and three groups of cracks were obtained, as shown in Figure 11. The ADI of the PZT1-PZT7 sensing path is significantly higher than that of other sensing paths after compensation. The sensing paths near the damage in the loose bolts exhibit higher ADIs. For the groups with crack damage, their ADIs not only have higher values but also increase as the cracks expand. The ADIs obtained by the algorithm are consistent with the actual signal variations.

### 3.3. Damage Imaging Results

**Figure 12:**
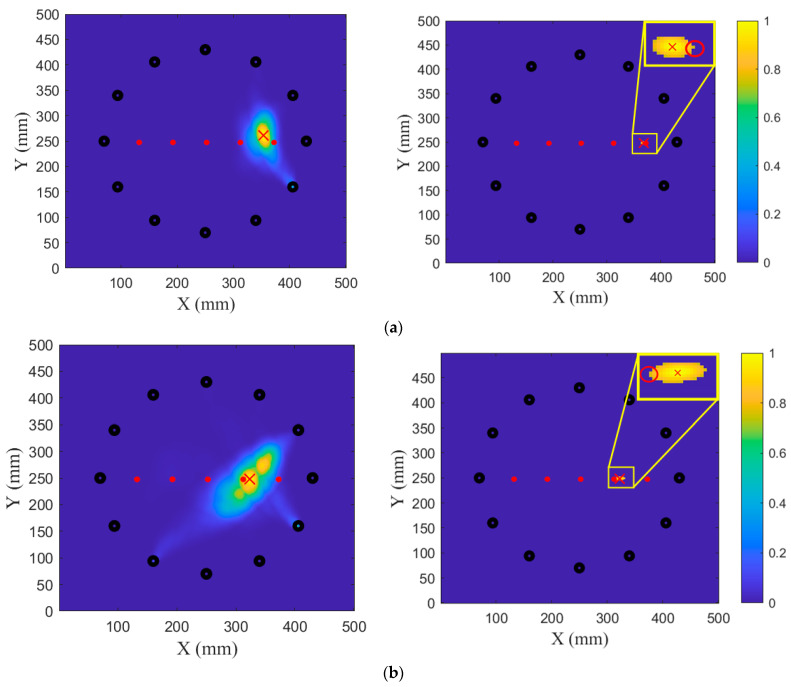
Imaging results of G1 and G2: (**a**) G1; (**b**) G2.

**Figure 13:**
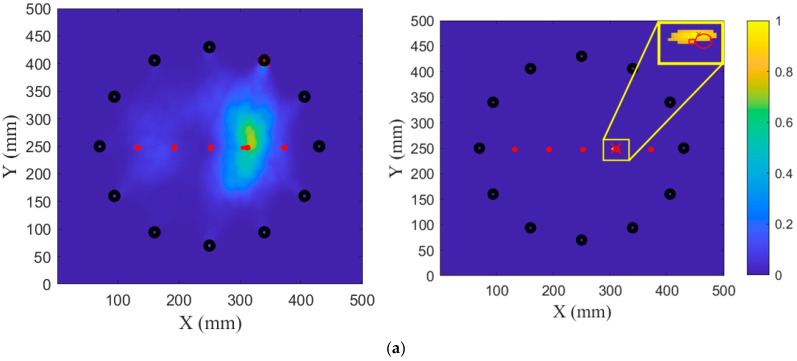

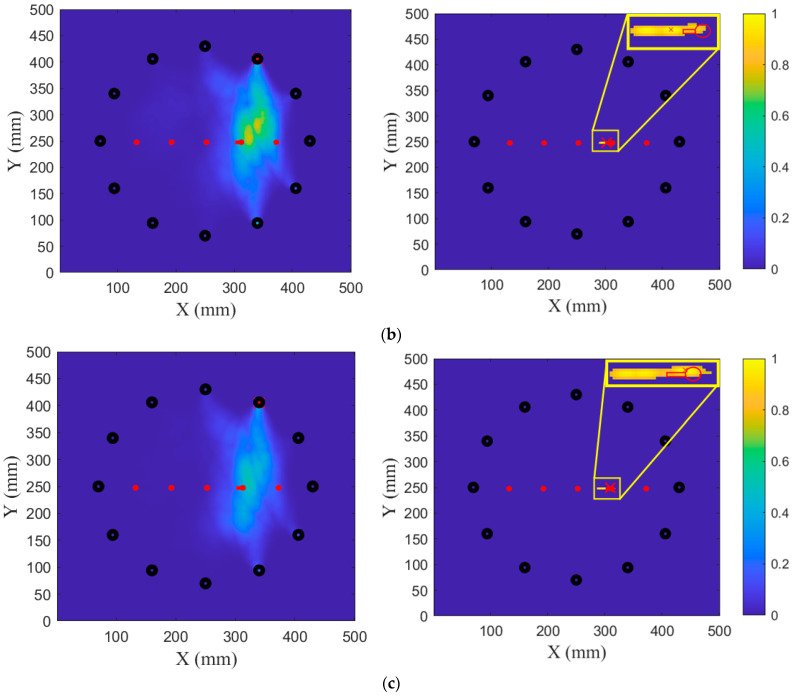
Imaging results of G3, G4 and G5: (**a**) G3; (**b**) G4; (**c**) G5.

The ADIs of all the sensing paths of the sensor array were calculated and are presented in the previous section. In this section, the damage imaging results of five groups of damage are presented, and the accuracy of damage location and imaging performance of the proposed method are evaluated in comparison with the localization results of the original RAPID algorithm. By combining the twelve PZTs in all possible non-repeating pairs, a total of 66 sensing paths are formed to construct the damage images. The imaging results of the original RAPID are shown on the left sides of Figure 12 and Figure 13, and the proposed method’s damage images are shown on the right sides of Figure 12 and Figure 13, where the maximum within the damage probability matrix is identified as the damage localization point, as indicated by a red cross. The actual damage is depicted in the image with a red overlay to facilitate a comparison of the damage shapes derived from the two methods. The red circles represent the actual position and coverage range of the screw hole. The red rectangles represent the actual length and width of the crack. In addition, red dots represent screw holes, and black dots represent PZTs.

Based on the maximum value of the probability of damage, the localization results of the original RAPID are at (354 mm, 262 mm) and (323 mm, 248 mm) for G1 and G2, respectively. The localization results of the proposed method are at (366 mm, 252 mm) and (318 mm, 249 mm), as shown in Figure 12.

Figure 13 illustrates the imaging results of cracks at 2 mm, 4 mm, and 6 mm. The original RAPID cannot obtain the approximate image result of the damage, and due to the overlap of sensing paths, the maximum value of the damage matrix appears near PZT3. The localization result is seriously deviated from the real damage location.

For G3, the proposed method yields a localization result at (308 mm, 251 mm). For G4, the localization result is at (294 mm, 252 mm). For G5, the localization result is at (293 mm, 252 mm). It can be concluded from the images that the method proposed in this paper can effectively locate a crack and its damage after expansion.

Table 2 gives the results of the localization of the original RAPID and the proposed method in this paper. The proposed method improves the location accuracy of loose bolts and reduces the error to less than 10 mm. For small cracks, the method proposed in this paper successfully locates the damage location, and the error is about 5 mm.

The localization error of G1 and G2 is 4 mm and 8 mm, respectively. For G3, the localization error is 2 mm compared to the actual crack coordinates at (305 mm, 250 mm). For G4, the localization error is 5 mm. For G5, the localization error is 5 mm. Analysis of the damage localization results indicates that the method’s localization error does not exceed 10 mm, and its localization accuracy is significantly higher than that of the original RAPID.

For loose bolts, the number of pixels (NOP) for G1 and G2 are 68 and 72, respectively. For crack growth, the number of pixels (NOP) for G3, G4, and G5 are 57, 93, and 114, respectively. The cracks in the damage image expand in the same horizontal direction as the actual cracks, and the damage degree also increases linearly. Consequently, it can be concluded that the method’s computational results under the same detection conditions are consistent with the actual situation. The proposed algorithm can quantify the degree of damage, and the results vary with the real damage.

## 4. Conclusions

In this paper, a density-clustering RAPID with an array-compensated damage index is proposed. By using an array-compensated damage index, a new probability distribution function and density clustering, the method can effectively quantify the loose bolts and cracks at the edge of a screw hole on a complex stiffened plate. The method proposed in this article enhances the original RAPID method by improving localization accuracy while retaining fast operation, and achieves quantitative diagnosis of damage comparable to other guided wave-based imaging algorithms.

However, while the proposed method can quantify the degree of crack growth, it is not able to directly estimate its actual length. Further research will focus on image processing for damage shape estimation, which would be able to achieve both the width and length estimation of the actual damage.

## Figures and Tables

**Table 1:** Calculation formula of DI [36].

Method Name	Mathematical Formula
FreAmpDiff	${DI}^{1}=\sqrt{\frac{\sum_{i=1}^{M/2}{\left|F_{MS,i}{-F}_{RS,i}\right|}^{2}}{\sum_{i=1}^{M/2}{\left|F_{RS,i}\right|}^{2}}}$
Cross Corr	${DI}^{2}=1-\frac{\sum \left({RS}_{i}-\bar{RS})\cdot ({MS}_{i}-\bar{MS}\right)}{sqrt(\left(\left(\sum {RS}^{2}-{\left(RS\right)}^{2}\right)\right)\cdot (\left(\sum {MS}^{2}-{\left(MS\right)}^{2}\right)))}$
RMSD	${DI}^{3}=\sqrt{\frac{\sum {\left({MS}_{i}-{RS}_{i}\right)}^{2}}{\sum {{RS}_{i}}^{2}}}$
FreDiff	${DI}^{4}=\frac{rms\left(RS\right)}{means\left(\left|RS\right|\right)}-\frac{rms\left(MS\right)}{means\left(\left|MS\right|\right)}$
PhaseDiff	${DI}^{5}=\sum {\left(\frac{{RS}_{i}}{\sqrt{\sum {{RS}_{i}}^{2}}}-\frac{{MS}_{i}}{\sum {{MS}_{i}}^{2}}\cdot \sum \left(\frac{{RS}_{i}\cdot {MS}_{i}}{\sqrt{\sum {{RS}_{i}}^{2}}}\right)\right)}^{2}$
CCD	${DI}^{6}=1-\frac{\sum \left(({RS}_{i}-\bar{RS})\cdot ({MS}_{i}-\bar{MS}\right))}{sqrt(\sum \left({\left({RS}_{i}-\bar{RS}\right)}^{2}\right)\cdot ({\left({MS}_{i}-\bar{MS}\right)}^{2}))}$
DiffCurveEnergy	${DI}^{7}=\frac{\sum diff{\left({MS}_{i}-{RS}_{i}\right)}^{2}}{\sum diff{\left({RS}_{i}\right)}^{2}}$
ScatAmp	${DI}^{8}=\frac{max\left|MS-RS\right|}{max(RS)}$
ChDiffSTD	${DI}^{9}=\left|\frac{std\left(MS\right)-std\left(RS\right)}{std\left(RS\right)}\right|$

**Table 2:** Results of proposed method (unit: mm).

Damage Type	Original RAPID		Proposed Method	
	Localization	Error	Localization	Error
G1(370, 250)	(354, 262)	20	(366, 252)	4
G2(310, 250)	(323, 248)	13	(318, 249)	8
G3(309, 250)	/	/	(311, 250)	2
G4(307, 250)	/	/	(302, 248)	5
G5(305, 250)	/	/	(310, 249)	5

## Data Availability

The data presented in this study are available on request from the corresponding author.
